# Small-Quantity Lipid-Based Nutrient Supplements Do Not Affect Plasma or Milk Retinol Concentrations Among Malawian Mothers, or Plasma Retinol Concentrations among Young Malawian or Ghanaian Children in Two Randomized Trials

**DOI:** 10.1093/jn/nxaa439

**Published:** 2021-02-09

**Authors:** Marjorie J Haskell, Rebecca Young, Seth Adu-Afaruwah, Anna Lartey, Harriet Eyram Teiko Okronipa, Kenneth Maleta, Ulla Ashorn, Josh M Jorgensen, Yue-Mei Fan, Charles D Arnold, Lindsay H Allen, Per Ashorn, Kathryn G Dewey

**Affiliations:** Institute for Global Nutrition, University of California, Davis, Davis, CA, USA; Department of Nutrition, University of California, Davis, Davis, CA, USA; Institute for Global Nutrition, University of California, Davis, Davis, CA, USA; Department of Nutrition, University of California, Davis, Davis, CA, USA; Department of Nutrition and Food Science, University of Ghana, Accra, Ghana; Department of Nutrition and Food Science, University of Ghana, Accra, Ghana; Institute for Global Nutrition, University of California, Davis, Davis, CA, USA; Department of Nutrition, University of California, Davis, Davis, CA, USA; Department of Nutrition and Food Science, University of Ghana, Accra, Ghana; Department of Public Health, Blantyre, School of Public Health and Family Medicine, College of Medicine, University of Malawi, Malawi; Center for Child Health Research, Faculty of Medicine and Health Technology, Tampere University, Tampere, Finland; Institute for Global Nutrition, University of California, Davis, Davis, CA, USA; Department of Nutrition, University of California, Davis, Davis, CA, USA; Center for Child Health Research, Faculty of Medicine and Health Technology, Tampere University, Tampere, Finland; Institute for Global Nutrition, University of California, Davis, Davis, CA, USA; Department of Nutrition, University of California, Davis, Davis, CA, USA; Institute for Global Nutrition, University of California, Davis, Davis, CA, USA; Department of Nutrition, University of California, Davis, Davis, CA, USA; USDA, ARS Western Human Nutrition Research Center, Davis, CA, USA; Center for Child Health Research, Faculty of Medicine and Health Technology, Tampere University, Tampere, Finland; Department of Paediatrics, Tampere University Hospital, Tampere, Finland; Institute for Global Nutrition, University of California, Davis, Davis, CA, USA; Department of Nutrition, University of California, Davis, Davis, CA, USA

**Keywords:** vitamin A, retinol, plasma, milk, lipid-based nutrient supplements, Ghana, Malawi

## Abstract

**Background:**

Vitamin A (VA) deficiency is prevalent in preschool-aged children in sub-Saharan Africa.

**Objectives:**

We assessed the effect of small-quantity lipid-based nutrient supplements (SQ-LNS) given to women during pregnancy and lactation and their children from 6 to 18 mo of age on women's plasma and milk retinol concentrations in Malawi, and children's plasma retinol concentration in Malawi and Ghana.

**Methods:**

Pregnant women (≤20 wk of gestation) were randomized to receive daily: *1*) iron and folic acid (IFA) during pregnancy only; *2*) multiple micronutrients (MMN; 800 μg retinol equivalent (RE)/capsule), or *3*) SQ-LNS (800 μg RE/20g) during pregnancy and the first 6 mo postpartum. Children of mothers in the SQ-LNS group received SQ-LNS (400 μg RE/20 g) from 6 to 18 mo of age; children of mothers in the IFA and MMN groups received no supplement. Plasma retinol was measured in mothers at ≤20 and 36 wk of gestation and 6 mo postpartum, and in children at 6 and 18 mo of age. Milk retinol was measured at 6 mo postpartum. VA status indicators were compared by group.

**Results:**

Among Malawian mothers, geometric mean (95% CI) plasma retinol concentrations at 36 wk of gestation and 6 mo postpartum were 0.97 μmol/L (0.94, 1.01 μmol/L) and 1.35 μmol/L (1.31, 1.39 μmol/L), respectively; geometric mean (95% CI) milk retinol concentration at 6 mo postpartum was 1.04 μmol/L (0.97, 1.13 μmol/L); results did not differ by intervention group. Geometric mean (95% CI) plasma retinol concentrations for Malawian children at 6 and 18 mo of age were 0.78 μmol/L (0.75, 0.81 μmol/L) and 0.81 μmol/L (0.78, 0.85 μmol/L), respectively, and for Ghanaian children they were 0.85 μmol/L (0.82, 0.88 μmol/L) and 0.88 μmol/L (0.85, 0.91 μmol/L), respectively; results did not differ by intervention group in either setting.

**Conclusions:**

SQ-LNS had no effect on VA status of mothers or children, possibly because of low responsiveness of the VA status indicators.

## Introduction

Vitamin A deficiency (VAD) is a public health problem among children <5 y of age in low- and middle-income countries, is associated with increased risk of diarrhea, respiratory diseases, and measles, and can lead to blindness and death (1). Although the global prevalence of VAD among children 6–59 mo of age declined from ∼39 to ∼29% between 1991 and 2013, the prevalence of VAD in sub-Saharan Africa remained unchanged at ∼48% (1). In 2017 the prevalence of VAD among Ghanaian children 6–59 mo of age was ∼21% nationally and ∼31% in the northern region (2). In Malawi, the national prevalence of VAD among children 6–59 mo of age was ∼22% in 2009 (3), but only ∼4% in 2015–2016 (4). However, the prevalence in 2015–2016 may have been underestimated because it was based on serum retinol-binding protein (RBP) concentrations <0.46 μmol/L, whereas much higher serum RBP cutoff values (<0.70 to <0.83 μmol/L) have been used in other surveys in the region, including in the 2009 survey in Malawi (3–6). Intervention programs to control VAD include the distribution of high-dose vitamin A capsules to children 6–59 mo of age, the fortification of staple foods, and the distribution of micronutrient powders to children 6–24 mo of age.

Small-quantity lipid-based nutrient supplements (SQ-LNS) were developed as an intervention to prevent undernutrition in pregnant and lactating women and young children (7). SQ-LNS are food-based supplements enriched with a vitamin and mineral complex to meet the needs of specific target populations, and can be eaten alone or added to foods prepared in households. They differ from multiple micronutrient (MMN) supplements in that they provide energy, protein, essential fatty acids, a greater number of micronutrients, and several macrominerals that are required for growth (7). Recent meta-analyses of SQ-LNS demonstrated reductions in adverse birth outcomes (8) and in child stunting, wasting, and anemia (9), but did not examine vitamin A status.

The International Lipid-based Nutrient Supplement (iLiNS) project included four randomized, controlled, community-based trials, two of which were designed to assess the effect of providing SQ-LNS to mothers during pregnancy and lactation, and to their children from 6 to 18 mo of age, on pregnancy outcomes, child growth and development, and other nutritional and health outcomes in Malawi and Ghana (https://ilins.ucdavis.edu/). As a subanalysis within these two iLiNS-DYAD trials in Malawi and Ghana, we assessed the effect of SQ-LNS on the vitamin A status of mother–child dyads in Malawi and young children in Ghana. The impact of SQ-LNS on the vitamin A status of mothers of Ghanaian children was reported separately, based on the effect of SQ-LNS on milk retinol concentration (10). The specific objectives of this analysis were *1*) to assess the effect of providing SQ-LNS to Malawian women during pregnancy and lactation on their plasma retinol concentrations at 36 wk of gestation and 6 mo postpartum, and milk retinol concentrations at 6 mo postpartum, and *2*) to assess the effect of the intervention on plasma retinol concentrations of children at 6 and 18 mo of age in both Malawi and Ghana.

## Methods

### Participants and study design

The participants in this subanalysis were selected randomly from the mother–child dyads who participated in the iLiNS trials in Malawi and Ghana, as described below. The Malawi trial was conducted in rural areas of the Mangochi district in southern Malawi between February 2011 and August 2012. The Ghana trial was conducted in semiurban areas of the Somanya–Odumasi–Kpong region, ∼70 km north of Accra, Ghana, between December 2009 and December 2011. The primary objective of both trials was to determine whether provision of SQ-LNS to women during pregnancy and the first 6 mo of lactation and to children from 6 to 18 mo of age improves fetal and child growth to a greater extent than consumption of iron and folic acid during pregnancy only, or an MMN capsule during pregnancy and the first 6 mo of lactation. The primary results of the trials and the trial design are described in detail elsewhere (11–13).

Women who visited the study clinics for antenatal care were eligible for the trials if they were at ≤20 wk of gestation (confirmed by ultrasound) and ≥18 y of age (Ghana) or >15 y of age (Malawi). Exclusion criteria for both trials were milk or peanut allergy, chronic disease requiring medical attention, pregnancy complications (moderate to severe edema, hemoglobin <50 g/L, systolic blood pressure >160 mmHg, or diastolic blood pressure >100 mmHg), intent to move away from the area, not a resident of the area, or unwillingness to take the study supplement; additional exclusion criteria in Ghana were HIV positivity (if indicated on the woman's antenatal card), asthma, and epilepsy. Women with twin pregnancies were eligible, and 1 twin from each pair was randomly selected for inclusion.

Informed consent was obtained from women for their participation and that of their infants upon delivery. The Institutional Review Board of the University of California, Davis, and the Ethics Committees of the Noguchi Memorial Institute for Medical Research, University of Ghana, and the Ghana Health Service approved the study protocol for the trial in Ghana. The Ethics Committees of the College of Medicine, University of Malawi, and the Pirkanmaa Hospital District, Finland, approved the study protocol for the trial in Malawi.

In both trials, pregnant women were randomized to one of three groups to receive: *1*) iron and folic acid (IFA, 60 mg iron and 400 μg folic acid as a capsule; standard of care) during pregnancy only; *2*) MMNs (800 μg retinol equivalent (RE) plus 17 micronutrients including 20 mg iron as a capsule) during pregnancy and the first 6 mo postpartum; or *3*) SQ-LNS (a 20-g sachet that contained 800 μg RE plus the same 17 micronutrients as the MMN capsule and 4 additional minerals: calcium, phosphorus, potassium, magnesium) during pregnancy and the first 6 mo postpartum. Women in the IFA group received a placebo capsule (200 mg calcium) during the first 6 mo postpartum. Children of mothers in the SQ-LNS group received SQ-LNS (a 20-g sachet containing 400 μg RE) from 6 to 18 mo of age; children in the IFA and MMN groups received no supplement. The IFA, MMN and placebo capsules were identical in appearance. Participants were provided with a 2-wk supply of their assigned capsule or SQ-LNS every 14 d by field workers, who delivered the supplements to participants’ homes. Adherence was assessed by maternal report and by counting any unused capsules or SQ-LNS sachets from the previous 2-wk period. The field coordinator and field workers who delivered supplements to participants were aware of the participants’ group assignments; all remaining staff, including anthropometrists and laboratory technicians, were masked to group assignments.

The IFA, MMN, and placebo capsules were manufactured by DSM Nutritional Products South Africa. The SQ-LNS was produced and packaged by Nutriset. Capsules and SQ-LNS were stored between 20 and 40°C at the field sites. The MMN capsules and 20-g SQ-LNS for women contained 800 μg RE, which is 100% of the FAO/WHO recommended safe intake during pregnancy and 95% of the recommended safe intake during lactation; the 20-g SQ-LNS for children contained 400 μg RE, which is 100% of the recommended safe intake (14). The nutrient composition of supplements is shown in **Supplemental Tables 1** and **2**.

### Collection of blood and milk samples

Nonfasting blood samples were collected from women at ≤20 and 36 wk of gestation and 6 mo postpartum and from children at 6 and 18 mo of age. Time of day and time of consumption of the last food other than tea or water were recorded. Blood was centrifuged at 2000 × *g* for 15 min at room temperature to obtain plasma, which was aliquoted into cryovials and stored at −80°C (Malawi) or −33 to −80°C (Ghana) for later analysis of the retinol concentration. At 6 mo postpartum, mothers in Malawi were asked to hand-express all milk from a single breast during a home visit. A sample (10 mL) of well-mixed milk was kept for later analysis of the retinol concentration. The remaining milk was returned to the mother to feed to her infant using a spoon. Milk samples were placed on ice packs, protected from light, and stored at −20°C within the first 24 h, and then at −80°C. Plasma and milk samples from Malawi and plasma samples from Ghana were shipped in dry ice to the University of California, Davis, for measurement of the retinol concentration.

### Laboratory analyses

Plasma and milk retinol concentrations were measured by HPLC (15, 16). A pooled plasma sample was analyzed in triplicate with each batch of study samples (plasma and milk) to assess the precision of the measurements; the intraday CV was ≤4.5% and interday CV was ≤2.3%. Control serum (SRM 968e, NIST) was analyzed 3 times to assess the accuracy of the retinol measurements; the measured retinol concentration was 1.65, 1.68, and 1.67 μmol/L; the certified value was 1.68 μmol/L. Plasma concentrations of C-reactive protein (CRP) and α_1_-acid glycoprotein (AGP) were measured by immunoassay using a COBAS Integra Analyzer (Roche Diagnostics). The cutoff values used for inadequate vitamin A status were: plasma retinol <0.7 μmol/L (children) (17), plasma retinol <1.05 μmol/L (women) (18), and milk retinol <1.05 μmol/L (17). Although it is known that plasma retinol concentration decreases during pregnancy, presumably because of hemodilution (19), we are not aware of a pregnancy-specific cutoff value for plasma retinol concentration. The cutoff of <1.05 μmol/L for milk retinol is based on an estimate of the amount of vitamin A intake from milk that is required for exclusively breastfed infants to meet their needs and build modest stores (20). The cutoff values used for biomarkers of inflammation were >5 mg/L for plasma CRP and >1 g/L for plasma AGP. Results for plasma retinol concentration are reported for Malawian mother–child pairs and for Ghanaian children. Milk retinol concentration is reported for Malawian mothers only; the effect of SQ-LNS on milk retinol concentrations for Ghanaian mothers was reported separately (10), and their plasma retinol concentrations were not measured.

### Sample size and statistical analysis

To calculate the sample size, we used a minimum effect size (Cohen's *d*) of 0.5 and assumed a 2-sided significance test with α = 0.05% and 80% power; this resulted in 79 per group (total *n* = 237). To account for attrition and participants missing a sample at any time point, 318 mother–child pairs were selected randomly in Malawi from mother–child dyads with all 5 biological samples (≤20 wk of gestation, 36 wk of gestation, 6 mo postpartum, child 6 mo and child 18 mo of age). In Ghana, there was an error in labeling of the IFA and MMN supplements that resulted in mixed exposure for some of the mother–child dyads (12); after excluding dyads with mixed exposure, 303 children were selected randomly from children with paired plasma samples (6 and 18 mo of age).

Distributions of outcome variables and key baseline variables were inspected for normality and transformed to natural logs, as necessary. Analyses were performed using SAS version 9.4. The covariates included in the ANCOVA models were obtained from the scientific literature and were preselected in our analysis plan. Covariates were included in the model if they were associated with the outcome variable (*P* < 0.1). Potential covariates for maternal plasma and milk retinol analyses included maternal estimated prepregnancy BMI (13), maternal education, maternal receipt of high-dose vitamin A capsule within 6–8 wk of giving birth, site of enrollment, season at enrollment, baseline household food insecurity score, baseline asset index, baseline plasma retinol concentration, and baseline HIV status. Potential covariates for child plasma retinol analyses included child receipt of high-dose vitamin A capsule, season at 18 mo of age, and all of the above except for baseline maternal HIV status and baseline maternal plasma retinol concentration, which were not included for Ghanaian children.

The primary statistical analysis was done by intention to treat. The difference in mean maternal plasma and milk retinol concentrations and the difference in mean child retinol concentration among the three intervention groups were tested with ANOVA (model without covariates) and ANCOVA (model with covariates) with *P* < 0.05 to indicate statistical significance. Post hoc pairwise comparisons of the intervention groups were done with the Tukey–Kramer test for ANOVA and ANCOVA *P* < 0.05 if the global null hypothesis was rejected with *P* < 0.05.

The proportion of women with low plasma and milk retinol concentrations and the proportion of children with low plasma retinol concentrations were compared between intervention groups using logistic regression. Pairwise comparisons between groups were done in the context of logistic regression if the global null hypothesis was rejected with *P* < 0.05.

A per protocol analysis was performed that included only participants whose adherence was high (**Supplemental Methods** and **Supplemental Results**).

Plasma and milk retinol concentrations may be altered during inflammation. We used correlation analysis to examine the relationship between plasma retinol concentration and plasma concentrations of CRP and AGP, separately, at the 36-wk gestation and 6-mo timepoints, and the relationship between milk retinol concentration and plasma concentrations of CRP and AGP, separately, at the 6-mo timepoint. If there was a statistically significant correlation (Spearman's correlation *P* < 0.1) between markers of inflammation and plasma retinol concentration or between markers of inflammation and milk retinol concentration, the method described by the BRINDA project (21) was used to correct the retinol results for inflammation. Both inflammation-corrected and noninflammation-corrected retinol results are presented.

Before applying the BRINDA correction, we first examined whether the intervention had a statistically significant effect on plasma concentrations of CRP or AGP at the 36-wk and 6-mo timepoints.

A sensitivity analysis was planned if results showed no difference in plasma or milk retinol concentrations among mothers between the SQ-LNS and MMN groups. For this analysis, these groups would be combined to assess whether there was a difference in plasma and/or milk retinol concentrations among women who received SQ-LNS/MMN compared with IFA. The same sensitivity analysis was planned for children at 6 mo of age if results showed no difference in plasma retinol concentrations between the SQ-LNS and MMN groups. Among children at 18 mo of age, a sensitivity analysis was planned if results showed no difference in plasma retinol concentrations between the MMN and IFA groups. For this analysis, these groups would be combined to assess whether there was a difference in plasma retinol concentration among children who received SQ-LNS compared with no supplement (combined MMN/IFA groups).

## Results

### Participants

In Malawi, 1391 women were enrolled in the trial, and 317 mother–child dyads were selected randomly for this subanalysis (**Supplemental Figure 1**). Among Malawian mothers, plasma retinol data were available for 313 women at 36 wk of gestation and for 316 women at 6 mo postpartum. Milk retinol data were available for 315 women at 6 mo postpartum. Among Malawian children, plasma retinol data were available for 254 children at 6 mo of age and for 253 children at 18 mo of age; some data were missing because there was insufficient plasma for the retinol analysis. In Ghana, 1320 mother–child dyads were enrolled in the trial; however, 510 were excluded from consideration for these analyses due to mixed exposure for these dyads (12). From the remaining 810 mother–child pairs, 303 children were selected randomly for the analysis of plasma retinol concentrations (**Supplemental Figure 2**). Plasma retinol data were available for 292 children at 6 and 18 mo of age.

Maternal characteristics at enrollment are shown in **Table 1**. Malawian mothers were ∼25 y of age, 20.9% were anemic, 11.2% were HIV+, 23.6% tested positive for malaria, and 46.8% had plasma retinol concentrations <1.05 μmol/L. Ghanaian mothers were ∼27 y of age, 12.6% were anemic, and 8.2% tested positive for malaria; as mentioned above, HIV+ women were excluded from the study; and plasma retinol concentration was not measured in Ghanaian mothers.

**TABLE 1:** Enrollment characteristics of Malawian and Ghanaian mothers

	Malawian mothers			Ghanaian mothers		
Characteristic	IFA	MMN	SQ-LNS	IFA	MMN	SQ-LNS
Number of participants	105	106	107	97	99	96
BMI, kg/m^2^	21.8 (2.6)	21.8 (2.7)	21.8 (2.9)	25.0 (5.5)	25.1 (4.6)	25.5 (5.4)
Age, y	25 (6)	25 (6)	25 (7)	28 (5)	27 (6)	28 (5)
Education, completed years at school	3.5 (3.3)	3.6 (3.4)	3.6 (3.5)	7.9 (3.5)	7.6 (3.4)	7.9 (3.6)
Proxy for socioeconomic status	−0.25 (0.83)	−0.18 (0.79)	−0.02 (0.86)	0.23 (0.89)	0.16 (0.79)	0.12 (0.88)
Plasma retinol concentration^1^, μmol/L	1.08 (0.27)	1.14 (0.27)	1.10 (0.32)	—	—	—
Plasma retinol <1.05 μmol/L, %	46.6	42.9	50.9	—	—	—
Inflammation–corrected plasma retinol <1.05 μmol/L, %	20.4	15.2	22.6	—	—	—
Anemic (hemoglobin <100 g/L), %	17.5	20.0	25.2	10.3	18.2	9.4
Primiparous, %	20.4	22.9	21.5	34.0	26.3	29.2
Low BMI (<18.5 kg/m^2^), %	5.8	3.8	9.3	2.1	2.1	2.2
Positive HIV test^2^, %	14.6	8.7	10.3	—	—	—
Positive malaria test (RDT), %	23.3	22.9	24.5	3.1	10.1	11.5

Values are mean (SD) or %. None of the characteristics differ by intervention group in either setting (*P* > 0.05). IFA, iron and folic acid; MMN, multiple micronutrients; RDT, rapid diagnostic test, SQ-LNS, small-quantity lipid-based nutrient supplements.

1 Plasma retinol concentration was not measured in Ghanaian women.

2 Ghanaian women were not tested for HIV.

### Inflammation

Within the subsample for these analyses, there were no significant differences in plasma CRP or AGP among intervention groups for Malawian mothers or Malawian or Ghanaian children. The prevalence of inflammation is presented in Supplementary Results.

### Maternal plasma retinol: Malawi

At 36 wk of gestation, the overall geometric mean (95% CI) plasma retinol concentration was 0.97 μmol/L (0.94, 1.01 μmol/L) and the prevalence of plasma retinol concentrations <1.05 μmol/L was 55%. After correcting for inflammation, the prevalence of low plasma retinol concentrations decreased to 31%. At 6 mo postpartum, the overall geometric mean (95% CI) plasma retinol concentration was 1.35 μmol/L (1.31, 1.39 μmol/L) and the prevalence of low plasma retinol concentrations was 13.9%, which decreased to 4.5% after correcting for inflammation. There were no differences in mean plasma retinol concentrations or the prevalence of low plasma retinol concentrations among the three intervention groups (**Table 2**), or between the combined SQ-LNS/MMN groups and the IFA group at either time point (**Supplemental Table 3**).

**TABLE 2:** Plasma and milk retinol concentrations of Malawian mothers at 36 wk of gestation and 6 mo postpartum by intervention group

							SQ-LNS vs. IFA: ratio of means or ORs (95% CI)	MMN vs. IFA: ratio of means or ORs (95% CI)	SQ-LNS vs. MMN: ratio of
						
	*n*			Geometric mean (95% CI)					means or ORs (95% CI)
Outcome^1^	IFA	MMN	SQ-LNS	IFA	MMN	SQ-LNS			
Plasma retinol, μmol/L									
36 wk of gestation	103	105	105	1.00 (0.94, 1.06)	0.99 (0.93, 1.04)	0.93 (0.87, 1.00)	0.93 (0.84, 1.04)	0.99 (0.89, 1.09)	0.95 (0.85, 1.05)
6 mo postpartum	103	106	107	1.40 (1.32, 1.45)	1.40 (1.30, 1.42)	1.30 (1.22, 1.38)	0.94 (0.86, 1.03)	0.98 (0.90, 1.07)	0.96 (0.88, 1.04)
Plasma retinol <1.05 μmol/L, %									
36 wk of gestation	103	105	105	50.5	53.3	65.7	1.88 (0.96, 3.68)	1.12 (0.58, 2.16)	1.68 (0.86, 3.23)
6 mo postpartum	103	106	107	11.7	11.3	20.6	1.96 (0.78, 4.91)	0.97 (0.35, 2.69)	2.03 (0.81, 5)
Inflammation-corrected plasma retinol, μmol/L									
36 wk of gestation	103	105	105	1.20 (1.15, 1.30)	1.20 (1.13, 1.27)	1.10 (1.07, 1.22)	0.94 (0.84, 1.04)	0.98 (0.89, 1.08)	0.95 (0.86, 1.06)
6 mo postpartum	103	106	106	1.60 (1.58, 1.73)	1.60 (1.55, 1.69)	1.60 (1.47, 1.65)	0.95 (0.87, 1.03)	0.98 (0.90, 1.07)	0.96 (0.89, 1.04)
Inflammation-corrected plasma retinol <1.05 μmol/L, %									
36 wk of gestation	103	105	105	31.1	27.6	34.3	1.16 (0.58, 2.32)	0.85 (0.41, 1.74)	1.37 (0.68, 2.78)
6 mo postpartum	103	106	107	3.9	1.9	8.4	2.27 (0.53, 9.73)	0.48 (0.06, 3.76)	4.78 (0.74, 33.3)
Milk retinol, μmol/L									
6 mo postpartum	103	106	106	1.00 (0.90, 1.22)	1.10 (0.94, 1.21)	1.00 (0.89, 1.15)	0.97 (0.77, 1.22)	1.02 (0.81, 1.28)	0.95 (0.76, 1.2)
Milk retinol <1.05 μmol/L, %									
6 mo postpartum	103	106	106	46.6	47.2	41.5	0.81 (0.42, 1.57)	1.02 (0.53, 1.97)	0.79 (0.41, 1.52)

1 None of the outcomes differed by intervention group. SQ-LNS, small-quantity lipid-based nutrient supplements; IFA, iron and folic acid; MMN, multiple micronutrients.

### Milk retinol: Malawi

At 6 mo postpartum the overall geometric mean (95% CI) milk retinol concentration was 1.04 μmol/L (0.97, 1.13 μmol/L) and the prevalence of milk retinol concentrations <1.05 μmol/L was 45.5%. Milk retinol concentration did not differ among the three groups (Table 2) or between the combined SQ-LNS/MMN and IFA groups (Supplemental Table 3). The median (p25, p75) time of day of milk collection was 07:30 (07:00, 08:00) and did not differ by group (*P* = 0.34). Milk retinol concentration was not correlated with maternal plasma retinol concentration (*r* = 0.028, *P* = 0.62), child plasma retinol concentration (*r* = −0.05, *P* = 0.39) or maternal plasma concentrations of CRP (*r* = 0.09, *P* = 0.13) or AGP (*r* = −0.01, *P* = 0.88).

### Child plasma retinol: Malawi

At 6 mo of age, the overall geometric mean (95% CI) plasma retinol concentration was 0.78 μmol/L (0.75, 0.81 μmol/L). The prevalence of plasma retinol concentrations <0.70 μmol/L was 22.0%, which decreased to 9.7% after correcting for inflammation. At 18 mo of age, the overall geometric mean (95% CI) plasma retinol concentration was 0.81 μmol/L (0.78, 0.85 μmol/L). The prevalence of low plasma retinol concentrations was 26.8%, which decreased to 9.2% after correcting for inflammation. There were no differences among the three groups (**Table 3**), between the combined SQ-LNS/MMN and IFA groups at 6 mo of age, or between the SQ-LNS and combined MMN/IFA groups at 18 mo of age (**Supplemental Table 4**). Child plasma retinol concentration at 6 mo of age was correlated with maternal plasma retinol concentration at enrollment and at 36 wk of gestation (*r* = 0.14; *P* < 0.03 for both maternal timepoints), but not at 6 mo postpartum (*r* = 0.09, *P* = 0.14). Child plasma retinol at 18 mo of age was correlated with maternal plasma retinol concentration at enrollment only (*r* = 0.14, *P* = 0.022).

**TABLE 3:** Plasma retinol concentrations of Malawian children at 6 and 18 mo of age by intervention group

									SQ-LNS vs. MMN: ratio of means or ORs (95% CI)
							SQ-LNS vs. IFA: ratio of means or ORs (95% CI)	MMN vs. IFA: ratio of means or ORs (95% CI)	
	*n*			Geometric mean (95% CI)					
Outcome^1^	IFA	MMN	SQ-LNS	IFA	MMN	SQ-LNS			
Plasma retinol, μmol/L									
6 mo of age	86	90	78	0.80 (0.74, 0.86)	0.80 (0.76, 0.86)	0.70 (0.68, 0.79)	0.92 (0.81, 1.04)	1.02 (0.90, 1.15)	0.90 (0.80, 1.02)
18 mo of age	85	90	78	0.80 (0.73, 0.85)	0.80 (0.77, 0.90)	0.80 (0.76, 0.88)	1.04 (0.91, 1.17)	1.06 (0.94, 1.20)	0.98 (0.87, 1.12)
Plasma retinol <0.70 μmol/L, %									
6 mo of age	86	90	78	27.9	30.0	39.7	1.70 (0.78, 3.74)	1.11 (0.51, 2.43)	1.54 (0.71, 3.33)
18 mo of age	85	90	78	28.2	25.6	26.9	0.94 (0.41, 2.14)	0.87 (0.39, 1.95)	1.07 (0.47, 2.44)
Inflammation-corrected plasma retinol, μmol/L									
6 mo of age	86	90	78	1.00 (0.94, 1.08)	1.00 (0.97, 1.09)	0.90 (0.87, 1.00)	0.93 (0.83, 1.04)	1.03 (0.92, 1.14)	0.91 (0.81, 1.01)
18 mo of age	85	90	78	1.00 (0.92, 1.05)	1.00 (0.96, 1.11)	1.00 (0.95, 1.08)	1.03 (0.92, 1.15)	1.05 (0.93, 1.17)	0.99 (0.88, 1.11)
Inflammation-corrected plasma retinol <0.70 μmol/L, %									
6 mo of age	86	90	78	9.3	7.8	12.8	1.43 (0.44, 4.69)	0.82 (0.23, 2.94)	1.74 (0.51, 5.88)
18 mo of age	85	90	78	9.4	6.7	10.3	1.10 (0.32, 3.81)	0.69 (0.18, 2.59)	1.60 (0.42, 5.88)

1 None of the outcomes differed by intervention group. SQ-LNS, small-quantity lipid-based nutrient supplements; IFA, iron and folic acid; MMN, multiple micronutrients.

### Child plasma retinol: Ghana

At 6 mo of age, the overall geometric mean (95% CI) plasma retinol concentration was 0.85 μmol/L (0.82, 0.88 μmol/L). The prevalence of plasma retinol concentrations <0.70 μmol/L was 25.6%, which decreased to 12.7% after correcting for inflammation. At 18 mo of age, the overall geometric mean (95% CI) plasma retinol concentration was 0.88 μmol/L (0.85, 0.91 μmol/L). The prevalence of low plasma retinol concentrations was 19.5%, which decreased to 6.5% after correcting for inflammation. There were no differences among the three groups (**Table 4**), between the combined SQ-LNS/MMN and IFA groups at 6 mo of age, or between the SQ-LNS and combined MMN/IFA groups at 18 mo of age (**Supplemental Table 5**).

**TABLE 4:** Plasma retinol concentrations of Ghanaian children at 6 and 18 mo of age by intervention group

									SQ-LNS vs. MMN: ratio of means or ORs (95% CI)
							SQ-LNS vs. IFA: ratio of means or ORs (95% CI)	MMN vs. IFA: ratio of means or ORs (95% CI)	
	*n*			Geometric mean (95% CI)					
Outcome^1^	IFA	MMN	SQ-LNS	IFA	MMN	SQ-LNS			
Plasma retinol, μmol/L									
6 mo of age	97	99	96	0.90 (0.83, 0.93)	0.80 (0.78, 0.87)	0.90 (0.81, 0.90)	0.97 (0.89, 1.07)	0.94 (0.86, 1.03)	1.03 (0.94, 1.14)
18 mo of age	97	99	96	0.90 (0.84, 0.96)	0.90 (0.82, 0.92)	0.90 (0.81, 0.92)	0.96 (0.87, 1.06)	0.96 (0.87, 1.06)	1.00 (0.90, 1.11)
Plasma retinol <0.70 μmol/L, %									
6 mo of age	97	99	96	18.6	33.3	25.0	1.46 (0.64, 3.35)	2.19 (0.99, 4.86)	0.67 (0.32, 1.41)
18 mo of age	97	99	96	14.4	19.2	25.0	1.98 (0.82, 4.76)	1.41 (0.57, 3.49)	1.40 (0.62, 3.23)
Inflammation-corrected plasma retinol, μmol/L									
6 mo of age	97	99	96	0.90 (0.90, 1.00)	0.90 (0.87, 0.97)	0.90 (0.88, 0.99)	0.99 (0.90, 1.07)	0.97 (0.89, 1.06)	1.01 (0.93, 1.11)
18 mo age	97	99	96	1.00 (0.98, 1.09)	1.00 (0.95, 1.06)	1.00 (0.97, 1.09)	0.99 (0.90, 1.08)	0.97 (0.89, 1.06)	1.02 (0.94, 1.12)
Inflammation-corrected plasma retinol <0.70 μmol/L, %									
6 mo of age	97	99	96	12.4	11.1	14.6	1.21 (0.45, 3.27)	0.89 (0.31, 2.52)	1.37 (0.50, 3.70)
18 mo of age	97	99	96	4.1	7.1	8.3	2.11 (0.48, 9.33)	1.77 (0.39, 8.06)	1.19 (0.34, 4.17)

1 None of the outcomes differed by intervention group. SQ-LNS, small-quantity lipid-based nutrient supplements; IFA, iron and folic acid; MMN, multiple micronutrients.

## Discussion

Provision of SQ-LNS to mothers during pregnancy and lactation and to their children from 6 to 18 mo of age did not affect plasma or milk retinol concentrations of Malawian mothers or plasma retinol concentrations of Malawian or Ghanaian children. The lack of effect on plasma concentrations may be due to low responsiveness of plasma retinol concentration for detecting an intervention effect in populations with a low prevalence of VAD (22, 23). Because plasma retinol concentration is under homeostatic regulation and is maintained within a narrow range when vitamin A status is adequate, it is not an optimal indicator for detecting change in status. Among Malawian mothers, the inflammation-corrected prevalence of VAD was ∼31% at 36 wk of gestation, but only ∼4.5% at 6 mo postpartum, suggesting that the high prevalence at 36 wk of gestation was related to hemodilution of pregnancy, as reported previously for women in Guinea Bissau and Bangladesh (24, 25).

The prevalence of low milk retinol concentrations among Malawian mothers was ∼46%, which was much higher than either the noninflammation-corrected or inflammation-corrected prevalence of low plasma retinol concentrations (13.9 and 4.5%, respectively). In a recent study among Zambian mothers, the prevalence of low milk retinol concentrations was also higher than the prevalence of low plasma retinol concentrations (54% and 34%, respectively) (26). In our study, milk retinol was not associated with plasma concentrations of CRP or AGP, the same as has been reported previously for Malawian women (27). The difference in estimates of prevalence of low milk compared with low plasma retinol concentrations may be related to the homeostatic control of plasma retinol, which limits the detection of marginal status, or possibly to milk sampling methods that may lead to underestimation of milk vitamin A concentration. Vitamin A is found in milk fat, and milk fat varies throughout the day and increases during a feeding episode. Milk was collected in the early morning, and the time of collection did not differ by group. However, milk collection was not standardized to time since the previous feeding episode, and this may have affected the retinol concentration. Also, incomplete expression of milk from the breast may result in lower milk vitamin A concentration because hind milk is highest in vitamin A. If milk is not well mixed prior to sampling for the retinol analysis, this could also affect the retinol concentration. For these reasons, it is helpful to express milk retinol per gram of milk fat in addition to per unit volume, which we were unable to do.

We would expect milk retinol concentration to be more responsive to daily consumption of SQ-LNS than plasma retinol concentration because milk retinol is not under homeostatic regulation and is derived directly from the maternal diet and from maternal stores (28). The lack of effect of SQ-LNS on milk retinol concentration may be related to the low prevalence of VAD, based on maternal plasma retinol concentrations at 6 mo postpartum. Although milk retinol is likely to increase among mothers with adequate status who receive a high-dose vitamin A supplement (29, 30), the increase in response to a modest daily dose of 800 μg RE/d would be harder to detect when milk retinol concentrations are adequate. The lack of effect may also be related to low responsiveness of milk retinol for detecting an intervention effect when it is not adjusted for milk fat. A study in Bangladesh showed that milk retinol expressed per gram of fat in casual samples was more responsive than serum retinol, modified relative dose response, or milk retinol in full-milk samples (expressed per unit volume or per gram of fat) for detecting an intervention effect (31). A recent study in Zambia showed that the prevalence of low milk retinol concentrations (∼54% at enrollment) decreased in response to 3 wk of supplementation with small daily doses of vitamin A (600 μg RE/d), compared with a control group, when milk retinol was expressed per gram of milk fat, but not when expressed per unit volume (26). Adjusting for milk fat is likely to reduce variability in milk retinol concentrations and increase the ability to detect an intervention effect. It is also possible that adherence to SQ-LNS was inadequate to increase milk retinol in our study, given that ∼64% of mothers reportedly consumed SQ-LNS on ≥70% of supplementation days during the first 6 mo of lactation (Supplementary Results).

Among Malawian children, the overall inflammation-corrected prevalence of VAD was <10% at 6 mo and 18 mo of age. However, because children with inflammation may have inadequate vitamin A status, the inflammation-corrected prevalence may underestimate the extent of the problem. Overall, 28% of children had elevated plasma CRP concentrations and 64% had elevated plasma AGP at 6 mo of age; 29% had elevated plasma CRP concentrations and 66% had elevated plasma AGP at 18 mo of age. The overall noninflammation-corrected prevalence of VAD was ∼22 and 27% at 6 and 18 mo of age, respectively. Using the WHO prevalence criteria for categorizing VAD as a public health concern (17), VAD would be considered a mild concern (prevalence of >2–10%) based on the inflammation-corrected prevalence of low plasma retinol concentration, but a severe concern (prevalence of ≥20%) based on noninflammation-corrected prevalence of low plasma retinol concentrations. Children in Malawi receive high-dose vitamin A capsules as per WHO recommendations (100,000 IU at 6–11 mo of age; 200,000 IU from 12 to 59 mo of age every 6 mo) (32), and this may contribute to the low inflammation-corrected prevalence of VAD. However, only 27% of children reportedly received their scheduled high-dose vitamin A capsule at 1 y of age and only 9% of mothers reportedly received a high-dose capsule (200 000 IU) within the first 8 wk after giving birth. Together, the plasma retinol results for mothers and children suggest that the prevalence of VAD was likely mild to moderate in the study population in Malawi, and this may account for the lack of effect of SQ-LNS on plasma retinol concentration. The observed prevalences of VAD are lower than those reported in the 2009 National Micronutrient Survey (3) (59.7% for children 6–11 mo of age; 89.9% for women 15–45 y of age) based on serum retinol concentrations <0.7 and <1.05 μmol/L, respectively. However, the observed prevalences are higher than those reported in the 2015–2016 National Micronutrient Survey (4) (∼4% among preschool children and 0.3% among women of reproductive age), based on serum RBP concentrations <0.46 μmol/L. However, because the serum molar ratio of retinol to RBP is ∼1:1 (33), we would expect the RBP cutoff to be similar to the cutoff of <0.70 for serum retinol. As mentioned above, the lower RBP cutoff may have underestimated the prevalence of VAD.

Among Ghanaian children, the overall inflammation-corrected prevalence of VAD was 12.6% at 6 mo of age and 7% at 18 mo of age. The overall noninflammation-corrected prevalence of VAD was 23.9% at 6 mo of age and 21% at 18 mo of age. Using the WHO prevalence criteria (17), VAD would be considered a mild (>2–10%) to moderate (≥10 to <20%) public health concern among Ghanaian children in our study population based on the inflammation-corrected prevalence of low plasma retinol concentrations, and a severe concern (≥20% prevalence) based on noninflammation-corrected prevalence of low plasma retinol concentrations. As in Malawi, children in Ghana receive high-dose vitamin A supplements from 6 to 59 mo age in accordance with the WHO guidelines (32) and 87% reportedly received a high-dose vitamin A capsule at 12 mo of age. Among Ghanaian mothers, 68% reportedly received a high-dose vitamin A capsule within the first 8 wk postpartum. The plasma retinol results in children and the previously reported low prevalence of low milk retinol concentrations among mothers suggest that VAD is mild to moderate in the Ghanaian study population, and this may explain the lack of effect of SQ-LNS on plasma retinol concentration.

In contrast to our results, provision of a MMN supplement containing 770 μg RE/d to Bangladeshi women, beginning early in pregnancy, resulted in higher mean plasma retinol concentration at 32 wk of gestation in comparison with women who received IFA (1.08 ± 0.33 compared with 0.99 ± 0.33 μmol/L) and a lower prevalence of plasma retinol concentrations <1.05 μmol/L (48.7 compared with 60.9%) (34). The amount of vitamin A provided by the supplement was similar to that provided by SQ-LNS in our study; however, the inflammation-corrected prevalence of plasma retinol concentrations <1.05 μmol/L in early pregnancy was higher among Bangladeshi women than among Malawian women (48 compared with 31%), and this may account for the difference in results. An earlier study in Malawi showed that supplementation of pregnant HIV+ women with 3 mg RE/d, beginning at ∼18–24 wk of gestation, resulted in a higher mean plasma retinol concentration at 38 wk of gestation compared with a control group (0.72 compared with 0.61 μmol/L) and a higher mean milk retinol concentration at 6 wk postpartum (1.32 compared with 0.95 μmol/L) (35). The observed effects on plasma and milk retinol concentrations are likely related to lower vitamin A status of the women compared with women in our study, and to the larger amount of vitamin A that was provided.

Studies have shown mixed results regarding the effect of SQ-LNS on child vitamin A status. Provision of 400 μg RE/d as SQ-LNS to Burkinabe children from 9 to 18 mo of age, along with treatment for diarrhea and malaria, resulted in a higher mean plasma RBP concentration at 18 mo of age compared with a nonintervention cohort; however, the prevalence of VAD remained high and did not differ between cohorts (50 compared with 57%) (36). A subanalysis of the WASH Benefits Study showed that among Kenyan children 6–24 mo of age with a high prevalence of VAD (∼53%), median serum RBP concentration was higher among children who received SQ-LNS than in a control group after 2 y of intervention, whereas SQ-LNS had no effect on serum RBP concentrations among Bangladeshi children of the same age with a low prevalence of VAD (∼17%) (37). In another study of Bangladeshi children, provision of SQ-LNS containing 200 μg RE/d from 6 to 11 mo of age and medium-quantity LNS containing 400 μg RE/d from 12 to 18 mo of age resulted in a higher mean plasma retinol concentration at 18 mo of age compared with children in a control group, but had no effect on the prevalence of VAD (∼8%) (38).

Similar to our findings, in populations in Madagascar and Bangladesh with a relatively low prevalence of VAD (∼13.7 and 13.0%, respectively), provision of LNS to mothers during pregnancy and lactation, and/or SQ-LNS to children, had no effect on serum RBP concentration or the prevalence of VAD among children 6–18 mo of age (Madagascar) or 6–24 mo of age (Bangladesh) (39, 40).

In summary, provision of SQ-LNS to Malawian mothers during pregnancy and lactation, and to Malawian and Ghanaian children from 6 to 18 mo of age did not affect plasma or milk retinol concentration in Malawian mothers or plasma retinol concentrations in Malawian and Ghanaian children. The lack of effect may be related to the mild to moderate prevalence of VAD among the study populations, and/or to the low responsiveness of plasma retinol concentration for detecting a change in status. Although the milk retinol results suggest a high prevalence of inadequate vitamin A status among mothers in Malawi, SQ-LNS had no effect on milk retinol concentration. The lack of adjustment for milk fat may have resulted in overestimation of the prevalence of low milk retinol concentrations, and/or reduced the responsiveness of milk retinol for detecting an intervention effect.

## Supplementary Material

nxaa439_Supplemental_Files

## Notes

Financial support for this work was provided by the Bill & Melinda Gates Foundation (Project Number OPP49817).

Author disclosures: The authors report no conflicts of interest.

Supplemental Figures 1 and 2, Supplemental Tables 1–5, Supplemental Methods, and Supplemental Results are available from the “Supplementary data” link in the online posting of the article and from the same link in the online table of contents at https://academic.oup.com/ajcn/.

Abbreviations used: AGP, α_1_-acid glycoprotein; CRP, C-reactive protein; IFA, iron and folic acid; MMN, multiple micronutrient; RBP, retinol-binding protein; RE, retinol equivalent; SQ-LNS, small-quantity lipid-based nutrient supplements; VA, vitamin A; VAD, vitamin A deficiency.

## References

[1] Stevens G , Bennett J, Hennocq Q, Lu Y, De-Regil L, Rogers L, Danaei G, Li G, White R, Flaxman S et al. Trends and mortality effects of vitamin A deficiency in children in 138 low-income and middle-income countries between 1991 and 2013: a pooled analysis of population-based surveys. Lancet Glob Health. 2015;3:e528–36. 26275329 10.1016/S2214-109X(15)00039-X

[2] University of Ghana, GroundWork, University of Wisconsin-Madison, KEMRI-Wellcome Trust, UNICEF. Ghana Micronutrient Survey. 2017. Accra, Ghana; 2017.

[3] National Statistics Office (NSO) . Malawi-National Micronutrient Survey 2009. Lilongwe (Malawi): Department of Nutrition, HIV and AIDS in the Office of President and Cabinet (DHNA-OPC); Ministry of Health (MOH); National Statistics Office (NSO); UNICEF; Centers for Disease Control (CDC); 2011.

[4] National Statistical Office (NSO), Community Health Sciences Unit (CHSU) [Malawi], Centers for Disease Control and Prevention (CDC), and Emory University. Malawi Micronutrient Survey 2015–16. Atlanta (GA): NSO, CHSU, CDC and Emory University, 2017.

[5] Food Security and Nutrition Analysis Unit. National Micronutrient and Anthropometric Nutrition Survey, Somalia 2009. Food Security and Nutrition Analysis Unit; WHO; UNICEF; World Food Programme (WFP); Center for International Health and Development (CIHD), Ministry of Health, Somalia; 2009.

[6] Uganda Bureau of Statistics (UBOS); ICF International. Uganda Demographic and Health Survey 2011—Addendum to Chapter 11; Uganda Bureau of Statistics. Kampala (Uganda): UBOS; and Calverton (MD): ICF International; 2012.

[7] Arimond M , Zeilani M, Jungjohann S, Brown K, Ashorn P, Allen L, Dewey K. Considerations in developing lipid-based nutrient supplements for prevention of undernutrition: experience from the International Lipid-Based Nutrient Supplements (iLiNS) project. Matern Child Nutr. 2015;11:31–61. 10.1111/mcn.12049 PMC6860325 23647784

[8] Das JK , Hoodbhoy Z, Salam RA, Bhutta AZ, Valenzuela-Rubio NG, Weise Prinzo Z, Bhutta ZA. Lipid-based nutrient supplements for maternal, birth, and infant developmental outcomes. Cochrane Database Syst Rev. 2018;8:CD012610–CD. 30168868 10.1002/14651858.CD012610.pub2 PMC6513224

[9] Das JK , Salam RA, Hadi YB, Sadiq Sheikh S, Bhutta AZ, Weise Prinzo Z, Bhutta ZA. Preventive lipid-based nutrient supplements given with complementary foods to infants and young children 6 to 23 months of age for health, nutrition, and developmental outcomes. Cochrane Database Syst Rev. 2019;5:CD012611. 31046132 10.1002/14651858.CD012611.pub3 PMC6497129

[10] Klevor MK , Haskell MJ, Lartey A, Adu-Afarwuah S, Zeilani M, Dewey KG. Lipid-based nutrient supplements providing approximately the recommended daily intake of vitamin A do not increase breast milk retinol concentrations among Ghanaian women. J Nutr. 2016;146:335–42. 26740682 10.3945/jn.115.217786

[11] Ashorn P , Alho L, Ashorn U, Cheung YB, Dewey KG, Gondwe A, Harjunmaa U, Lartey A, Phiri N, Phiri TE et al. Supplementation of maternal diets during pregnancy and for 6 months postpartum and infant diets thereafter with small-quantity lipid-based nutrient supplements does not promote child growth by 18 months of age in rural Malawi: a randomized controlled trial. J Nutr. 2015;145:1345–53. 25926413 10.3945/jn.114.207225

[12] Adu-Afarwuah S , Lartey A, Okronipa H, Ashorn P, Zeilani M, Peerson JM, Arimond M, Vosti S, Dewey KG. Lipid-based nutrient supplement increases the birth size of infants of primiparous women in Ghana. Am J Clin Nutr. 2015;101:835–46. 25833980 10.3945/ajcn.114.091546

[13] Adu-Afarwuah S , Lartey A, Okronipa H, Ashorn P, Peerson JM, Arimond M, Ashorn U, Zeilani M, Vosti S, Dewey KG. Small-quantity, lipid-based nutrient supplements provided to women during pregnancy and 6 mo postpartum and to their infants from 6 mo of age increase the mean attained length of 18-mo-old children in semi-urban Ghana: a randomized controlled trial. Am J Clin Nutr. 2016;104:797–808. 27534634 10.3945/ajcn.116.134692 PMC4997301

[14] Food and Agriculture Organization (FAO) and World Health Organization (WHO). Vitamin and mineral requirements in human nutrition. Rome: FAO; 2004.

[15] Bieri JG , Tolliver TJ, Catignani GL. Simultaneous determination of alpha-tocopherol and retinol in plasma or red cells by high pressure liquid chromatography. Am J Clin Nutr. 1979;32:2143–9. 484533 10.1093/ajcn/32.10.2143

[16] Turner T , Burri BJ. Rapid isocratic HPLC method and sample extraction procedures for measuring carotenoid, retinoid, and tocopherol concentrations in human blood and breast milk for intervention studies. Chromatographia. 2012;75:241–52.

[17] World Health Organization. Indicators for assessing vitamin A deficiency and their application in monitoring and evaluating intervention programmes. Geneva (Switzerland): WHO/UNICEF; 1996.

[18] Sommer A , Davidson F. Assessment and control of vitamin A deficiency: the Annecy Accords. J Nutr. 2002;132:2845S–50S. 12221259 10.1093/jn/132.9.2845S

[19] Bates C . Vitamin A in pregnancy and lactation. Proc Nutr Soc. 1983;42:65–79. 6340124 10.1079/pns19830008

[20] Allen L , Haskell M. Vitamin A requirements of infants under 6 months. Food Nutr Bull. 2001;22:214–34.

[21] Larson LM , Guo J, Williams AM, Young MF, Ismaily S, Addo OY, Thurnham D, Tanumihardjo SA, Suchdev PS, Northrop-Clewes CA. Approaches to assess vitamin A status in settings of inflammation: Biomarkers Reflecting Inflammation and Nutritional Determinants of Anemia (BRINDA) project. Nutrients. 2018 Aug 16;10. 10.3390/nu10081100 PMC6115742 30115830

[22] Olson JA . Serum levels of vitamin A and carotenoids as reflectors of nutritional status. J Natl Cancer Inst. 1984;73:1439–44. 6439934

[23] Tanumihardjo S , Russell R, Stephensen C, Gannon B, Craft N, Haskell M, Lietz G, Schulze K, Raiten D. Biomarkers of Nutrition for Development (BOND)—vitamin A review. J Nutr. 2016;146:1816S–48S. 27511929 10.3945/jn.115.229708 PMC4997277

[24] Kaestel P , Martinussen T, Aaby P, Michaelsen KF, Friis H. Serum retinol is associated with stage of pregnancy and the acute phase response in pregnant women in Guinea-Bissau. J Nutr. 2012;142:942–7. 22437561 10.3945/jn.111.155937

[25] Lee V , Ahmed F, Wada S, Ahmed T, Ahmed AS, Parvin Banu C, Akhter N. Extent of vitamin A deficiency among rural pregnant women in Bangladesh. Public Health Nutr. 2008;11:1326–31. 18547455 10.1017/S1368980008002723

[26] Palmer AC , Chileshe J, Hall AG, Barffour MA, Molobeka N, West KP Jr, Haskell MJ. Short-term daily consumption of provitamin A carotenoid-biofortified maize has limited impact on breast milk retinol concentrations in Zambian women enrolled in a randomized controlled feeding trial. J Nutr. 2016;146:1783–92. 27466608 10.3945/jn.116.233700

[27] Dancheck B , Nussenblatt V, Kumwenda N, Lema V, Neville MC, Broadhead R, Taha TE, Ricks MO, Semba RD. Status of carotenoids, vitamin A, and vitamin E in the mother-infant dyad and anthropometric status of infants in Malawi. J Health Popul Nutr. 2005;23:343–50. 16599105

[28] Cabezuelo MT , Zaragozá R, Barber T, Viña JR. Role of vitamin A in mammary gland development and lactation. Nutrients. 2019;12:80. 10.3390/nu12010080 PMC7019238 31892157

[29] Bezerra DS , de Araújo KF, Azevêdo GMM, Dimenstein R. A randomized trial evaluating the effect of 2 regimens of maternal vitamin A supplementation on breast milk retinol levels. J Hum Lact. 2010;26:148–56. 20110563 10.1177/0890334409356445

[30] Dror DK , Allen LH. Retinol-to-fat ratio and retinol concentration in human milk show similar time trends and associations with maternal factors at the population level: a systematic review and meta-analysis. Adv Nutr. 2018;9:332S–46S. 29846525 10.1093/advances/nmy021 PMC6008956

[31] Rice AL , Stoltzfus RJ, de Francisco A, Kjolhede CJ. Evaluation of serum retinol, the modified-relative-dose-response ratio, and breast-milk vitamin A as indicators of response to postpartum maternal vitamin A supplementation. Am J Clin Nutr. 2000;71:799–806. 10702176 10.1093/ajcn/71.3.799

[32] World Health Organization (WHO). Guideline: vitamin A supplementation for infants and children 6–59 months of age. Geneva: WHO; 2011. 24575452

[33] Gamble MV , Ramakrishnan R, Palafox NA, Briand K, Berglund L, Blaner WS. Retinol binding protein as a surrogate measure for serum retinol: studies in vitamin A-deficient children from the Republic of the Marshall Islands. Am J Clin Nutr. 2001;73:594–601. 11237937 10.1093/ajcn/73.3.594

[34] Schulze KJ , Mehra S, Shaikh S, Ali H, Shamim AA, Wu LS, Mitra M, Arguello MA, Kmush B, Sungpuag P et al. Antenatal multiple micronutrient supplementation compared to iron-folic acid affects micronutrient status but does not eliminate deficiencies in a randomized controlled trial among pregnant women of rural Bangladesh. J Nutr. 2019;149(7):1260. 31006806 10.1093/jn/nxz046 PMC6602890

[35] Semba RD , Kumwenda N, Taha TE, Mtimavalye L, Broadhead R, Miotti PG, Eisinger W, Hoover D, Chiphangwi JD. Plasma and breast milk vitamin A as indicators of vitamin A status in pregnant women. Int J Vitam Nutr Res. 2000;70:271–7. 11214351 10.1024/0300-9831.70.6.271

[36] Abbeddou S , Yakes Jimenez E, Some JW, Ouedraogo JB, Brown KH, Hess SY. Small-quantity lipid-based nutrient supplements containing different amounts of zinc along with diarrhea and malaria treatment increase iron and vitamin A status and reduce anemia prevalence, but do not affect zinc status in young Burkinabe children: a cluster-randomized trial. BMC Pediatr. 2017;17:46. 28152989 10.1186/s12887-016-0765-9 PMC5288861

[37] Stewart CP , Dewey KG, Lin A, Pickering AJ, Byrd KA, Jannat K, Ali S, Rao G, Dentz HN, Kiprotich M et al. Effects of lipid-based nutrient supplements and infant and young child feeding counseling with or without improved water, sanitation, and hygiene (WASH) on anemia and micronutrient status: results from 2 cluster-randomized trials in Kenya and Bangladesh. Am J Clin Nutr. 2019;109:148–64. 30624600 10.1093/ajcn/nqy239 PMC6358037

[38] Campbell RK , Shaikh S, Schulze K, Arguello M, Ali H, Wu L, West KP Jr, Christian P. Micronutrient and inflammation status following one year of complementary food supplementation in 18-month-old rural Bangladeshi children: a randomized controlled trial. Nutrients. 2020;12(5):1452. 10.3390/nu12051452 PMC7284655 32443412

[39] Stewart CP , Fernald LCH, Weber AM, Arnold C, Galasso E. Lipid-based nutrient supplementation reduces child anemia and increases micronutrient status in Madagascar: a multiarm cluster-randomized controlled trial. J Nutr. 2020;150:958–66. 32006028 10.1093/jn/nxz320 PMC7138674

[40] Dewey KG , Mridha M, Matias SL, Cummins JR, Arnold CD, Young RT, Maalouf-Manasseh Z, Vosti SA. Effectiveness of home fortification with lipid-based nutrient supplements (LNS) or micronutrient powder on child growth, development, micronutrient status, and health expenditures in Bangladesh. Washington (DC): FHI 360/Food and Nutrition Technical Assistance III Project (FANTA); 2017.

