# Supplementary material for: Small-Quantity Lipid-Based Nutrient Supplements Do Not Affect Plasma or Milk Retinol Concentrations Among Malawian Mothers, or Plasma Retinol Concentrations among Young Malawian or Ghanaian Children in Two Randomized Trials
Source: J Nutr. 2021 Feb 9;151(4):1029–37. doi: 10.1093/jn/nxaa439 (PMC8030706; doi:10.1093/jn/nxaa439)
Supplement: nxaa439_Supplemental_Files [file nxaa439_supplemental_files.zip › Supplemental Figure 1 Dec 8 2020.pptx]

## Slide 1
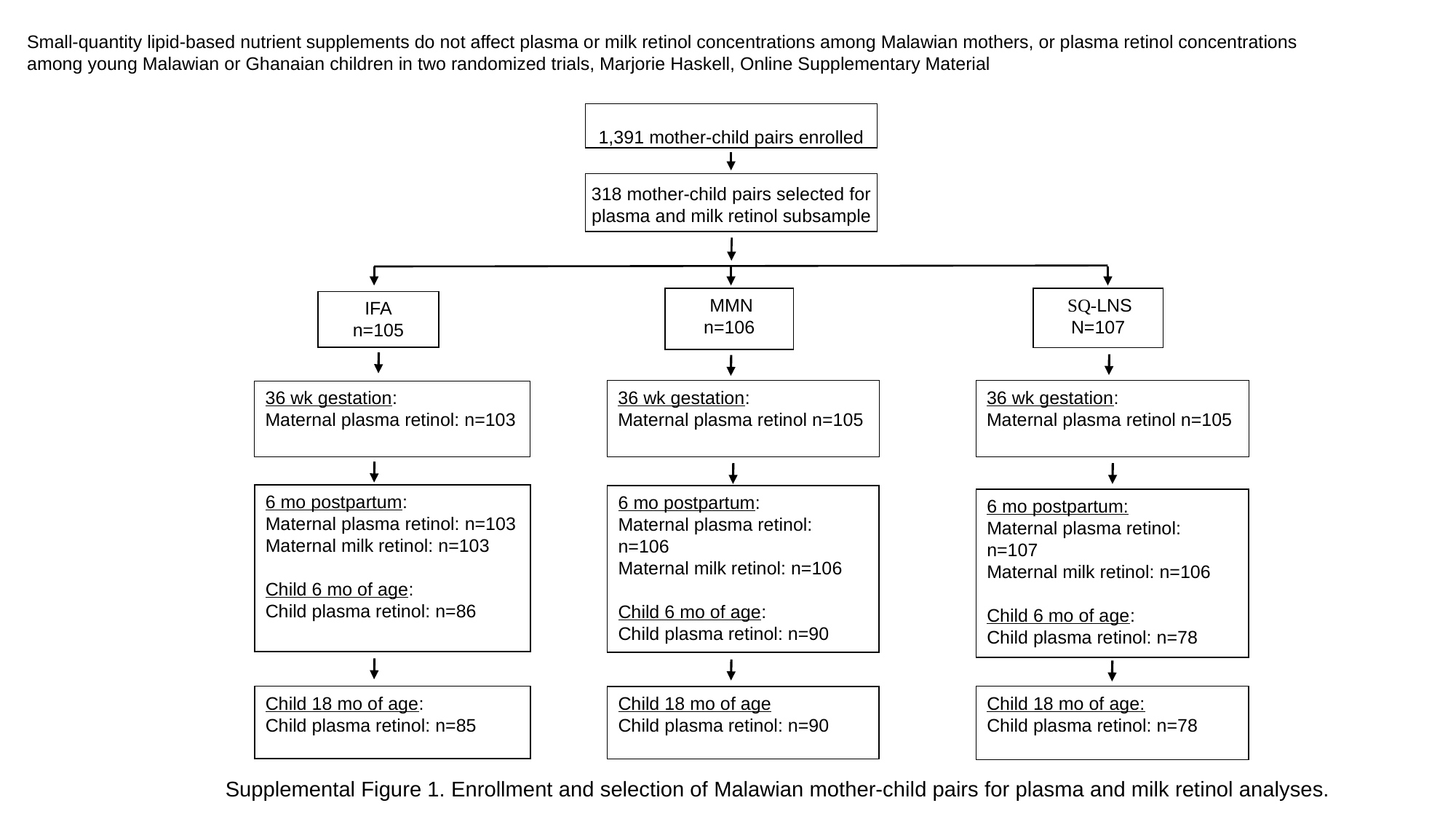

Small-quantity lipid-based nutrient supplements do not affect plasma or milk retinol concentrations among Malawian mothers, or plasma retinol concentrations
among young Malawian or Ghanaian children in two randomized trials, Marjorie Haskell, Online Supplementary Material
1,391 mother-child pairs enrolled
318 mother-child pairs selected for plasma and milk retinol subsample
 MMN
n=106
 SQ-LNS
N=107
36 wk gestation:
Maternal plasma retinol n=105
36 wk gestation:
Maternal plasma retinol n=105
36 wk gestation:
Maternal plasma retinol: n=103
6 mo postpartum:
Maternal plasma retinol: n=103
Maternal milk retinol: n=103
Child 6 mo of age:
Child plasma retinol: n=86
6 mo postpartum:
Maternal plasma retinol: n=106
Maternal milk retinol: n=106
Child 6 mo of age:
Child plasma retinol: n=90
6 mo postpartum:
Maternal plasma retinol: n=107
Maternal milk retinol: n=106
Child 6 mo of age:
Child plasma retinol: n=78
Child 18 mo of age:
Child plasma retinol: n=85
Child 18 mo of age
Child plasma retinol: n=90
Child 18 mo of age:
Child plasma retinol: n=78
IFA
n=105
Supplemental Figure 1. Enrollment and selection of Malawian mother-child pairs for plasma and milk retinol analyses.
