# Supplementary material for: Small-Quantity Lipid-Based Nutrient Supplements Do Not Affect Plasma or Milk Retinol Concentrations Among Malawian Mothers, or Plasma Retinol Concentrations among Young Malawian or Ghanaian Children in Two Randomized Trials
Source: J Nutr. 2021 Feb 9;151(4):1029–37. doi: 10.1093/jn/nxaa439 (PMC8030706; doi:10.1093/jn/nxaa439)
Supplement: nxaa439_Supplemental_Files [file nxaa439_supplemental_files.zip › Supplemental Figure 2 Dec 8 2020.pptx]

## Slide 1
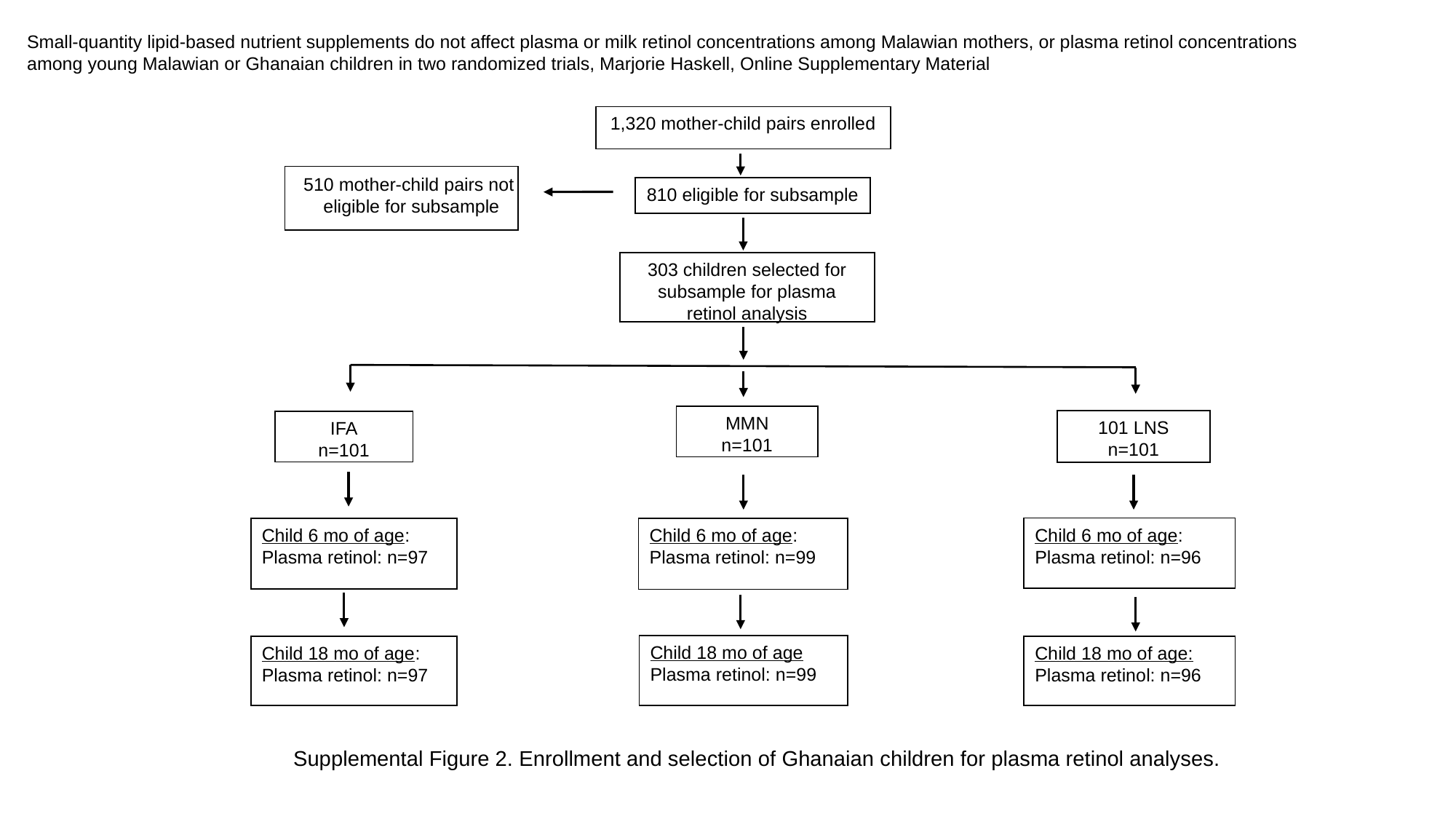

Small-quantity lipid-based nutrient supplements do not affect plasma or milk retinol concentrations among Malawian mothers, or plasma retinol concentrations
among young Malawian or Ghanaian children in two randomized trials, Marjorie Haskell, Online Supplementary Material
1,320 mother-child pairs enrolled
510 mother-child pairs not
 eligible for subsample
810 eligible for subsample
303 children selected for subsample for plasma retinol analysis
MMN
n=101
101 LNS
n=101
IFA
n=101
Child 6 mo of age:
Plasma retinol: n=96
Child 6 mo of age:
Plasma retinol: n=97
Child 6 mo of age:
Plasma retinol: n=99
Child 18 mo of age
Plasma retinol: n=99
Child 18 mo of age:
Plasma retinol: n=97
Child 18 mo of age:
Plasma retinol: n=96
Supplemental Figure 2. Enrollment and selection of Ghanaian children for plasma retinol analyses.
