# Supplementary material for: Small-Quantity Lipid-Based Nutrient Supplements Do Not Affect Plasma or Milk Retinol Concentrations Among Malawian Mothers, or Plasma Retinol Concentrations among Young Malawian or Ghanaian Children in Two Randomized Trials
Source: J Nutr. 2021 Feb 9;151(4):1029–37. doi: 10.1093/jn/nxaa439 (PMC8030706; doi:10.1093/jn/nxaa439)
Supplement: nxaa439_Supplemental_Files [file nxaa439_supplemental_files.zip › Supplementary Material Dec 2 2020.docx]

|  |  |  |  |
| --- | --- | --- | --- |

**Supplementary Methods**

*Statistical analyses*. A per protocol analysis was performed that included only participants whose adherence was high, defined as consumption of supplements on ≥70% of supplementation days during pregnancy and lactation for mothers.

**Supplementary Results**

*Inflammation*. At enrollment, the prevalence of plasma CRP >5 mg/L among Malawian and Ghanaian mothers was 38.3% and 35.9%, respectively; the prevalence of AGP >1 g/L was lower, 8.4 and 5.8%, respectively. Among Malawian mothers, the prevalence of elevated plasma CRP at 36 wk gestation and 6 mo postpartum was 28.4% and 11.8% respectively; the prevalence of elevated plasma AGP was 5.8% and 24.1% respectively. Among Malawian children, the prevalence of elevated plasma CRP at 6 mo and 18 mo of age was 28.1% and 29.3%, respectively, and the prevalence of elevated plasma AGP at 6 mo and 18 mo of age was 64.4% and 65.6%. Among Ghanaian children, the prevalence of elevated plasma CRP at 6 and 18 mo of age was 12.7% and 18.8% respectively, and the prevalence of elevated plasma AGP at 6 mo and 18 mo of age was 30.2% and 43.5%.

*Per protocol analysis***.** As mentioned above, good adherence was defined as reported consumption of supplements on ≥70% of supplementation days. Among Malawian participants in this subsample analysis, 74% of mothers had good adherence during pregnancy, and 64% of mothers had good adherence during the first 6 mo postpartum. Among Ghanaian participants, 76% of mothers had good adherence during the first 6 mo postpartum. In the per protocol analysis, among Malawian children at 6 mo of age, the geometric mean (95% CI) inflammation-corrected plasma retinol concentration was lower in the combined SQ-LNS/MMN groups compared to the IFA group, 0.9 (0.87, 1) µmol/L vs. 1.02 (0.97, 1.06) µmol/L respectively, n=163). There were no other differences in results between the per protocol and intention-to-treat analysis.

|  |  |  |  |
| --- | --- | --- | --- |
|  |  |  |  |
| **Supplementary Tables**  Supplemental Table 1. Composition of supplements given to pregnant and lactating women ^1^ | | | |
|  | | | |
|  | | | |
|  | | | |
|  | | | |
|  | | | |
|  | | | |

| Nutrient | IFA | MMN | LNS |  |
| --- | --- | --- | --- | --- |
| Ration, g/d | 1 tablet | 1 tablet | 20 |  |
| Total energy, kcal |  |  | 118 |  |
| Protein, g |  |  | 2.6 |  |
| Fat, g |  |  | 10 |  |
| Linoleic acid, g |  |  | 4.59 |  |
| α-Linolenic acid, g |  |  | 0.59 |  |
| Vitamin A, *µ*g RE |  | 800 | 800 |  |
| Vitamin C, mg |  | 100 | 100 |  |
| Vitamin B-1, mg |  | 2.8 | 2.8 |  |
| Vitamin B-2, mg |  | 2.8 | 2.8 |  |
| Niacin, mg |  | 36 | 36 |  |
| Folic acid, µg | 400 | 400 | 400 |  |
| Pantothenic acid, mg |  | 7 | 7 |  |
| Vitamin B-6, mg |  | 3.8 | 3.8 |  |
| Vitamin B-12, *µ*g |  | 5.2 | 5.2 |  |
| Vitamin D, IU |  | 400 | 400 |  |
| Vitamin E, mg |  | 20 | 20 |  |
| Vitamin K, *µ*g |  | 45 | 45 |  |
| Iron, mg | 60 | 20 | 20 |  |
| Zinc, mg |  | 30 | 30 |  |
| Copper, mg |  | 4 | 4 |  |
| Calcium, mg |  |  | 280 |  |
| Phosphorus, mg |  |  | 190 |  |
| Potassium, mg |  |  | 200 |  |
| Magnesium, mg |  |  | 65 |  |
| Selenium, *µ*g |  | 130 | 130 |  |
| Iodine, *µ*g |  | 250 | 250 |  |
| Manganese,mg |  | 2.6 | 2.6 |  |
| IFA, iron folic acid tablet; MMN, multiple micronutrient tablet; SQ-LNS, small-quantity  lipid-based nutrient supplements  ^1^ nutrient composition includes nutrients from ingredients and from the premix | | | | |

**Supplementary Tables**

Supplemental Table 2. Composition of supplements given to young children ^1^

| Nutrient | MMN | SQ-LNS |
| --- | --- | --- |
| Ration, g/d | 1 tablet | 20 |
| Total energy, kcal |  | 118 |
| Protein, g |  | 2.6 |
| Fat, g |  | 9.6 |
| Linoleic acid, g |  | 4.46 |
| α-Linolenic acid, g |  | 0.58 |
| Vitamin A, *µ*g RE | 400 | 400 |
| Vitamin C, mg | 30 | 30 |
| Vitamin B-1, mg | 0.3 | 0.3 |
| Vitamin B-2, mg | 0.4 | 0.4 |
| Niacin, mg | 4 | 4 |
| Folic acid, *µ*g | 80 | 80 |
| Pantothenic acid, mg | 1.8 | 1.8 |
| Vitamin B-6, mg | 0.3 | 0.3 |
| Vitamin B-12, *µ*g | 0.5 | 0.5 |
| Vitamin D, *µ*g | 5 | 5 |
| Vitamin E, mg | 6 | 6 |
| Vitamin K, *µ*g | 30 | 30 |
| Iron, mg | 6 | 6 |
| Zinc, mg | 8 | 8 |
| Copper, mg | 0.34 | 0.34 |
| Calcium, mg | 280 | 280 |
| Phosphorous, mg | 190 | 190 |
| Potassium, mg | 200 | 200 |
| Magnesium, mg | 40 | 40 |
| Selenium, *µ*g | 20 | 20 |
| Iodine, *µ*g | 90 | 90 |
| Manganese, mg | 1.2 | 1.2 |

MMN, micronutrient supplements; SQ-LNS, small quantity lipid-based nutrient

supplements

^1^ nutrient composition includes nutrients from ingredients and the premix

|  |
| --- |

| Supplemental Table 3. Plasma and milk retinol concentrations of Malawian mothers at 36 wk gestation and 6 mo postpartum by intervention group – 2-group comparison | | | | | | | | | |  |
| --- | --- | --- | --- | --- | --- | --- | --- | --- | --- | --- |
|  | |  | |  | |  | |  | |  |
| Outcome ^1^ | N | | | | Geometric mean (95% CI) | | | | Ratio of means or percentages  (95% CI) | |
|  | SQ-LNS/MMN | | IFA | | SQ-LNS/MMN | | IFA | |  |  |
| Plasma retinol, *µ*mol/L |  | | | | | | | | | |
| 36 wk gestation | 210 | | 103 | | 0.96 (0.92, 1.00) | | 1.00 (0.94, 1.06) | | 0.96 (0.89, 1.04) | |
| 6 mo postpartum | 213 | | 103 | | 1.33 (1.28, 1.38) | | 1.38 (1.32, 1.45) | | 0.96 (0.90, 1.03) | |
| Plasma retinol <1.05 *µ*mol/L, % |  | | | | | | | | | |
| 36 wk gestation | 210 | | 103 | | 125 (59.5) | | 52 (50.5) | | 1.44 (0.90, 2.32) | |
| 6 mo postpartum | 213 | | 103 | | 34 (16.0) | | 12 (11.7) | | 1.44 (0.71, 2.92) | |
| Inflammation-corrected plasma retinol, *µ*mol/L |  | | | | | | | | | |
| 36 wk gestation | 210 | | 103 | | 1.17 (1.12, 1.22) | | 1.22 (1.15, 1.30) | | 0.96 (0.89, 1.03) | |
| 6 mo postpartum | 213 | | 103 | | 1.59 (1.53, 1.65) | | 1.65 (1.58, 1.73) | | 0.96 (0.9, 1.02) | |
| Inflammation-corrected plasma retinol <1.05 *µ*mol/L, % |  | | | | | | | | | |
| 36 wk gestation | 210 | | 103 | | 65 (31.0) | | 32 (31.1) | | 0.99 (0.60, 1.66) | |
| 6 mo postpartum | 213 | | 103 | | 11 (5.1) | | 4 (3.9) | | 1.35 (0.42, 4.36) | |
| Milk retinol, *µ*mol/L |  | | | | | | | | | |
| 6 mo postpartum | 212 | | 103 | | 1.04 (0.95, 1.14) | | 1.05 (0.9, 1.22) | | 0.99 (0.84, 1.17) | |
| Milk retinol <1.05 *µ*mol/L, % |  | | | | | | | | | |
| 6 mo postpartum | 212 | | 103 | | 94 (44.3) | | 48 (46.6) | | 0.91 (0.57, 1.47) | |
| ^1^ None of the outcomes differed by intervention group. IFA, iron folic acid; MMN, multiple micronutrients; SQ-LNS, small-quantity lipid-based nutrient supplements.   \| Supplemental Table 4. Plasma retinol concentrations among Malawian children at 6 mo and 18 mo of age by group –  2-group comparison \| \| \| \| \| \| \| --- \| --- \| --- \| --- \| --- \| --- \| \|  \|  \|  \|  \|  \|  \| \| Outcome ^1^ \|  \|  \|  \|  \| Ratio of means or percentages  (95% CI) \| \| N \| \| Geometric mean (95% CI) \| \| \| Supp \| No Supp \| Supp \| No Supp \| \| Plasma retinol, *µ*mol/L \|  \|  \|  \|  \|  \| \| 6 mo of age ^2^ \| 168 \| 86 \| 0.77 (0.68, 0.79) \| 0.79 (0.76, 0.84) \| 0.97 (0.89, 1.06) \| \| 18 mo of age ^3^ \| 78 \| 175 \| 0.82 (0.76, 0.88) \| 0.81 (0.77, 0.85) \| 1.01 (0.92, 1.11) \| \| Plasma retinol <1.05 *µ*mol/L, %  6 mo of age ^2^ \| 168 \| 86 \| 58 (16.0) \| 24 (28.0) \| 1.36 (0.77, 2.41) \| \| 18 mo of age ^3^ \| 78 \| 175 \| 21 (26.9) \| 47 (26.9) \| 1.00 (0.55, 1.84) \| \| Inflammation-corrected plasma retinol, *µ*mol/L \|  \|  \|  \|  \|  \| \| 6 mo of age ^2^ \| 168 \| 86 \| 0.98 (0.87, 1.00) \| 1.00 (0.97, 1.06) \| 0.98 (0.9, 1.06) \| \| 18 mo of age ^3^ \| 78 \| 175 \| 1.02 (0.95, 1.08) \| 1.01 (0.96, 1.06) \| 1.01 (0.92, 1.09) \| \| Inflammation-corrected plasma retinol <0.70 *µ*mol/L, % \|  \|  \|  \|  \|  \| \| 6 mo of age ^2^ \| 168 \| 86 \| 17 (10.1) \| 16 (9.3) \| 1.10 (0.45, 2.67) \| \| 18 mo of age ^3^ \| 78 \| 175 \| 8 (10.3) \| 14 (8.0) \| 1.31 (0.53, 3.29) \| | | | | | | | | | |  |

^1^ None of the outcomes differed by intervention group. ^2^ At 6 mo of age, Supp = combined SQ-LNS/MMN groups and

No Supp = IFA group; ^3^ At 18 mo of age, Supp = SQ-LNS group and No Supp = combined MMN/IFA groups; IFA, iron folic acid;

MMN, multiple micronutrients; SQ-LNS, small-quantity lipid-based nutrient supplements.

| Supplemental Table 5. Plasma retinol concentrations among Ghanaian children at 6 mo and 18 mo of age by group –  2-group comparison | | | | | |
| --- | --- | --- | --- | --- | --- |
|  |  |  |  |  |  |
| Outcome ^1^ |  |  |  |  | Ratio of means or percentages  (95% CI) |
|  | N | | Geometric mean (95% CI) | |  |
|  | Supp | No Supp | Supp | No Supp |  |
| Plasma retinol, *µ*mol/L |  |  |  |  |  |
| 6 mo of age ^2^ | 195 | 97 | 0.84 (0.81, 0.87) | 0.88 (0.83, 0.93) | 0.96 (0.90, 1.02) |
| 18 mo of age ^3^ | 96 | 196 | 0.86 (0.83, 0.90) | 0.88 (0.84, 0.96) | 0.98 (0.90, 1.05) |
|  |  |  |  |  |  |
| Plasma retinol <1.05 *µ*mol/L, %  6 mo of age ^2^ | 195 | 97 | 57 (29.2) | 36 (18.6) | 1.81 (0.99, 3.30) |
| 18 mo of age ^3^ | 96 | 196 | 24 (25.0) | 33 (16.9) | 1.65 (0.91, 2.99) |
| Inflammation-corrected plasma retinol, *µ*mol/L |  |  |  |  |  |
| 6 mo of age ^2^ | 195 | 97 | 0.93 (0.89, 0.96) | 0.95 (0.9, 1.00) | 0.98 (0.91, 1.04) |
| 18 mo of age ^3^ | 96 | 196 | 1.03 (0.98, 1.05) | 1.02 (0.98, 1.09) | 1.01 (0.94, 1.07) |
| Inflammation-corrected plasma retinol <0.70 *µ*mol/L, % |  |  |  |  |  |
| 6 mo of age ^2^ | 195 | 97 | 25 (12.8) | 12 (12.4) | 1.04 (0.50, 2.18) |
| 18 mo of age ^3^ | 96 | 196 | 8 (8.3) | 11 (5.6) | 1.53 (0.59, 3.95) |

^1^ None of the outcomes differed by intervention group. ^2^ At 6 mo of age, Supp = combined SQ-LNS/MMN groups and

No Supp = IFA group; ^3^ At 18 mo of age, Supp = SQ-LNS group and No Supp = combined MMN/IFA groups; IFA,

iron folic acid; MMN, multiple micronutrients; SQ-LNS, small-quantity lipid-based nutrient supplements.
